# Editorial Correspondence

**Published:** 1871-09

**Authors:** W. F. Westmoreland

**Affiliations:** New York


### Editorial and Miscellaneous.

### EDITORIAL CORRESPONDENCE.

New York, September 15th, 1871.

*Dear Journal,*—I arrived in this city four days ago, and since my arrival, although very unwell, have been hard at work. I find many of the prominent medical men still absent from the city, and those that have returned from their annual tours of recreation are not yet regularly in the harness for the next year's drudgery.

The medical schools and hospitals are not yet under way for the winter's work—the few medical students now in the city are called together in the lecture rooms of the different schools by an occasional clinical lecture—it is evident, then, what difficulties I have to contend with in getting at the current medical news of the city.

My first visit after my arrival was to that *great* and *good* man, Dr. J. Marion Sims, who had only a few days before returned from Europe, this time bringing with him his interesting family, who, for the past nine years have made their home in Paris. New York will, in the future, be his permanent home, and this side of the Atlantic the field of his labors.

Every American should feel proud of Dr. Sims, as he is the only medical man who, by his genius and learning, has so impressed the professional men of the medical centers of Europe, as none, however distinguished, are too great to do him honor.

Dr. Sims looks in better health than I have ever seen him; and although near sixty, does not look fifty years of age. His long-asked for and long-looked for new edition of Uterine Surgery, revised, with additions, etc., will appear next year.

He has now ready for the press an illustrated monograph upon the etiology and treatment of "Trismus Náscentium," which will appear some time during the winter. Many will recollect Dr. Sim's articles upon Trismus Náscentium, published in the "American Journal of Medical Sciences," 1846 and 1847, and recollect, too, that his views were not accepted by the profession. He publishes this monograph that his views may be preserved and be more acceptable to the profession, and not with the idea that they will *at present* meet with any better reception by the profession than they did years ago. He feels, however, confident of their correctness, and that at some future period, however distant, his views will be accepted.

He takes the position that trismus náscentium is the result of a displacement of the occipital bone inwards, compressing the brain, or rather the medulla oblongata; and that in every case of trismus we have the parietal bones at the lambdoidal suture over-riding or elevated above the occipital bone; that is, the occipital bone is depressed. He claims that all that is necessary to relieve the little sufferer is to restore the occipital bone to its normal position, which may be readily done by placing the infant on its side, thus removing all pressure from the occipital bone.

Until he did me the honor of reading that portion of his manuscript giving the facts upon which he bases his etiology of trismus in infants, I, like most of his friends, and certainly the mass of the profession, regarded this theory as one of the mistakes of his early youth; but after hearing his facts and carefully examining the reports of several cases so promptly relieved by his plan of treatment—which is simply position—and others where all the symptoms could be relieved, or induced at will by depressing or elevating the occipital bone, I was forced to admit that the subject required a more serious consideration than had been given it by the profession.

I to-day visited Bellevue Hospital with my friend Dr. J. W. S. Gouley, one of the surgeons to this immense charity. On one of the cases the Doctor expected to have performed his favorite operation, lithotripsy, but found the urethra and bladder too irritable to admit readily his lithotriptor. The other case had submitted to two sittings, but a sufficient length of time had not elapsed from the last sitting to even examine the case. He has performed lithotripsy in a number of cases with very flattering success.

As compensation for my disappointment he operated for stricture of the urethra. He does not make a hobby of any one of the operations for stricture, but performs alike, when indicated, internal and external urethrotomy, divulsion, etc. In this case he performed the operation of divulsion, with Sir Henry Thompson's Divulsor, or Dilator, as improved by himself. The operation was performed without anesthetics of any kind, and with much less suffering than I expected to have seen. A No. 14 (English,) metallic bougie, readily followed the divulsor. The divulsion could have as readily been made to receive a No. 18 bougie, but in this case it was not thought necessary.

Dr. Gouley is one of the rising surgeons of this city, and I predict for him a brilliant future. With a good mind, untiring energy, and a devotion to his profession rarely equaled, he is now commencing to reap the reward of his labors.

For the past several years he has given special attention to the diseases of the genito-urinary organs, and has now a work nearly ready for the press, entitled "Clinical Lectures on Diseases of the Genito-Urinary Organs," which will appear early next year.

To-day I had the pleasure of a call from Dr. T. Gaillard Thomas. He looks in the most perfect health, and not at all reduced by the arduous labors of the past few years. In addition to a laborious city and consultation practice, Dr. Thomas, during the exercises in the College of Physicians and Surgeons—in which he is the Professor of the Diseases of Women—lectures six times a week; and in addition to all this, has found time within the past year to re-write, with extended additions, his most excellent work on the diseases of females, so well known and so highly appreciated in the South. None of these facts do I get from Dr. Thomas, as he is extremely modest and retiring. The Doctor has the enviable reputation of being the best teacher in the city. He informed me, to-day, that the manuscript for his new edition was in the hands of the publisher, and that the work would appear in a few months.

Since my arrival here I have made repeated inquiries of physicians and surgeons, with whom I have come in contact, in regard to the results of electrolysis in the treatment of malignant affections, and more particularly in regard to Dr. Neftel, whose reported results have produced some sensation in certain localities of Georgia and Alabama. But few profess to know much about Dr. Neftel's treatment by electrolysis, the majority conveying the idea, by a shrug of the shoulders, that they have but little confidence in his results. I called at his rooms last evening, and saw him make an application to a cancerous breast in a lady from the South. I found Dr. Neftel exceedingly affable and communicative, but with all, to my surprise, rather modest and retiring. He feels that he is developing, but not yet perfected, an important therapeutic agent in malignant affections. In a few words, he claims that malignant growths are dependent upon the development of the malignant or cancerous cells; but the disease is at first local in character, but after a time becomes constitutional. That when local, before the lymphatics become involved, electrolysis will arrest the development and disperse the malignant growth, and relieve the patient much more permanently than can possibly be done with the knife; more effectually, he contends, from the fact that by electrolysis we can destroy cells in a large area—cells that are often left after the amputation of cancerous masses. I intend to see more of this before I leave the city, and will then be better prepared to give my impressions upon the subject. The mere admission that the electrical current will disperse or destroy malignant growths is an advance not to be ignored.

I have several times had the pleasure of meeting our talented young townsman, Dr. C. A. Simpson. The Doctor is giving his entire attention to the diseases of the eye and ear. He proposes making a specialty of the eye and ear. He is now with Dr. Roosa and staff, where he has any amount of material. The Doctor will remain in New York during the winter, and has promised to furnish the "JOURNAL" with clinical reports from the Eye and Ear Infirmaries.

Devotedly, W. F. WESTMORELAND.

